# An Exploratory Single-Cell Analysis Identifies Candidate Shared Molecular Features in Proliferative Diabetic Retinopathy and Parkinson’s Disease

**DOI:** 10.3390/genes17091004

**Published:** 2026-08-26

**Authors:** Xinting Wang, Siqi Zhou, Ning Yang, Xinrong Xu

**Affiliations:** Department of Ophthalmology, Nanjing University of Chinese Medicine, Nanjing 210023, China; xintinghappy1017@sina.com (X.W.);

**Keywords:** diabetic retinopathy, Parkinson’s disease, cell–cell communication, ITGB2, retina–brain axis

## Abstract

**Background**: Diabetic retinopathy (DR) and Parkinson’s disease (PD) are prevalent, progressive disorders that cause irreversible visual impairment and motor dysfunction, respectively. Increasing evidence suggests that retinal alterations may precede and predict neurodegeneration in PD, indicating potential shared pathogenic mechanisms. This study aimed to delineate the molecular and cellular connections between DR and PD, with a particular focus on their convergent neuroimmune and vascular pathways. **Methods**: We integrated single-cell RNA sequencing (scRNA-seq) datasets derived from proliferative DR (PDR) retinal fibrovascular membranes and PD brain tissues. Cell-type-associated transcriptional features were identified within each dataset, and corresponding cell populations were compared to identify candidate overlapping molecular features. CellChat was used to infer potential ligand–receptor interactions between cell populations. Gene set enrichment analysis (GSEA) and gene set variation analysis (GSVA) were performed to explore associated biological pathways and transcriptional programs. **Results**: A2M, NRP1, and ETS2 were identified as candidate shared transcriptional features in corresponding microglial and endothelial cell populations across the PDR and PD datasets. CellChat analysis predicted an ITGB2–ICAM1 (integrin beta-2-Intercellular Adhesion Molecule 1) ligand–receptor interaction in both datasets, although the predicted sender–receiver relationships differed according to tissue context. GSEA and GSVA identified overlapping inflammatory, apoptotic, hypoxia-related, and epithelial–mesenchymal transition-associated transcriptional programs across the analyzed datasets. These findings suggest potentially convergent immune–vascular and inflammatory features between PDR and PD. **Conclusions**: This exploratory single-cell analysis identified candidate cell-type-associated transcriptional features and potential cell–cell communication patterns shared between PDR and PD. The predicted ITGB2–ICAM1 interaction provides a hypothesis for further investigation of immune–vascular communication.

## 1. Introduction

Diabetic retinopathy (DR) and Parkinson’s disease (PD) represent two major chronic disorders that profoundly impact ocular and neurological health, collectively contributing to a substantial global health burden. DR, a progressive microvascular complication of diabetes mellitus, is recognized as the leading cause of preventable blindness among working-age adults worldwide [1]. The prevalence of DR is closely paralleling the rising incidence of diabetes in both developed and developing regions [2,3]. The natural history of DR involves a transition from early, often asymptomatic stages to more advanced forms such as proliferative DR (PDR), which are characterized by neovascularization and a significantly increased risk of vision loss [4]. On the other hand, PD is the second most prevalent neurodegenerative disorder [5]. PD predominantly affects individuals over the age of 60 and is marked by progressive motor and non-motor dysfunctions resulting from dopaminergic neuronal loss in the substantia nigra [6,7]. Both diseases not only diminish patients’ quality of life but also impose immense socioeconomic costs due to long-term disability and increased healthcare utilization.

Despite advances in clinical care, the therapeutic landscape for DR and PD remains suboptimal, with considerable unmet needs. The management of DR relies heavily on regular ophthalmologic surveillance and interventions, such as pan-retinal laser photocoagulation and intravitreal injections of anti-vascular endothelial growth factor (anti-VEGF) agents for vision-threatening complications [8,9,10]. These treatments are often limited by incomplete efficacy, recurrence, and the requirement for repeated procedures. Similarly, the current mainstay of PD therapy centers on dopamine replacement strategies, including levodopa and dopamine agonists [11]. Although effective in controlling motor symptoms, these treatments are frequently associated with motor fluctuations, dyskinesias, and a spectrum of non-motor adverse effects over time [12]. Thus, neither disease-modifying nor curative therapies have emerged for either DR or PD, underscoring the necessity to elucidate shared pathogenic mechanisms that might inform novel intervention strategies.

Recent research has begun to unravel intriguing epidemiological and mechanistic interrelationships between DR and neurodegenerative conditions such as PD. Epidemiological surveys suggest that individuals with diabetes, particularly those with advanced DR, exhibit an elevated risk of developing PD and other neurodegenerative diseases [13,14]. At the molecular and cellular levels, both DR and PD are characterized by chronic inflammation, microvascular dysfunction, mitophagy, oxidative stress, and neurodegeneration, suggesting a convergence of pathophysiological pathways [15,16]. Notably, the retina and the brain share similar embryological origins and structural features, including a blood–tissue barrier and complex neurovascular units, which may render them susceptible to analogous insults in the context of metabolic and neurodegenerative disorder [17]. Furthermore, by combining liquid biopsy proteomics with artificial intelligence, it was discovered that retinal degeneration occurs in PD, and the cells that drive DR changed with the progression of the disease [18]. These insights prompt further investigation into the cellular crosstalk and molecular signatures underpinning both diseases, which could provide a foundation for the development of cross-disease biomarkers and therapeutic targets.

However, despite emerging evidence for shared pathogenic mechanisms, the precise nature of cellular interactions and intercellular signaling events linking DR and PD remains insufficiently characterized. Most prior investigations have relied on bulk transcriptomic analyses or animal models, which lack the resolution to dissect cell-type-specific contributions and dynamic cellular states within the affected tissues. Furthermore, the complexity of the retina and brain neurovascular units necessitates high-dimensional approaches to resolve cell-specific gene expression changes at single-cell resolution.

**Figure 1:**
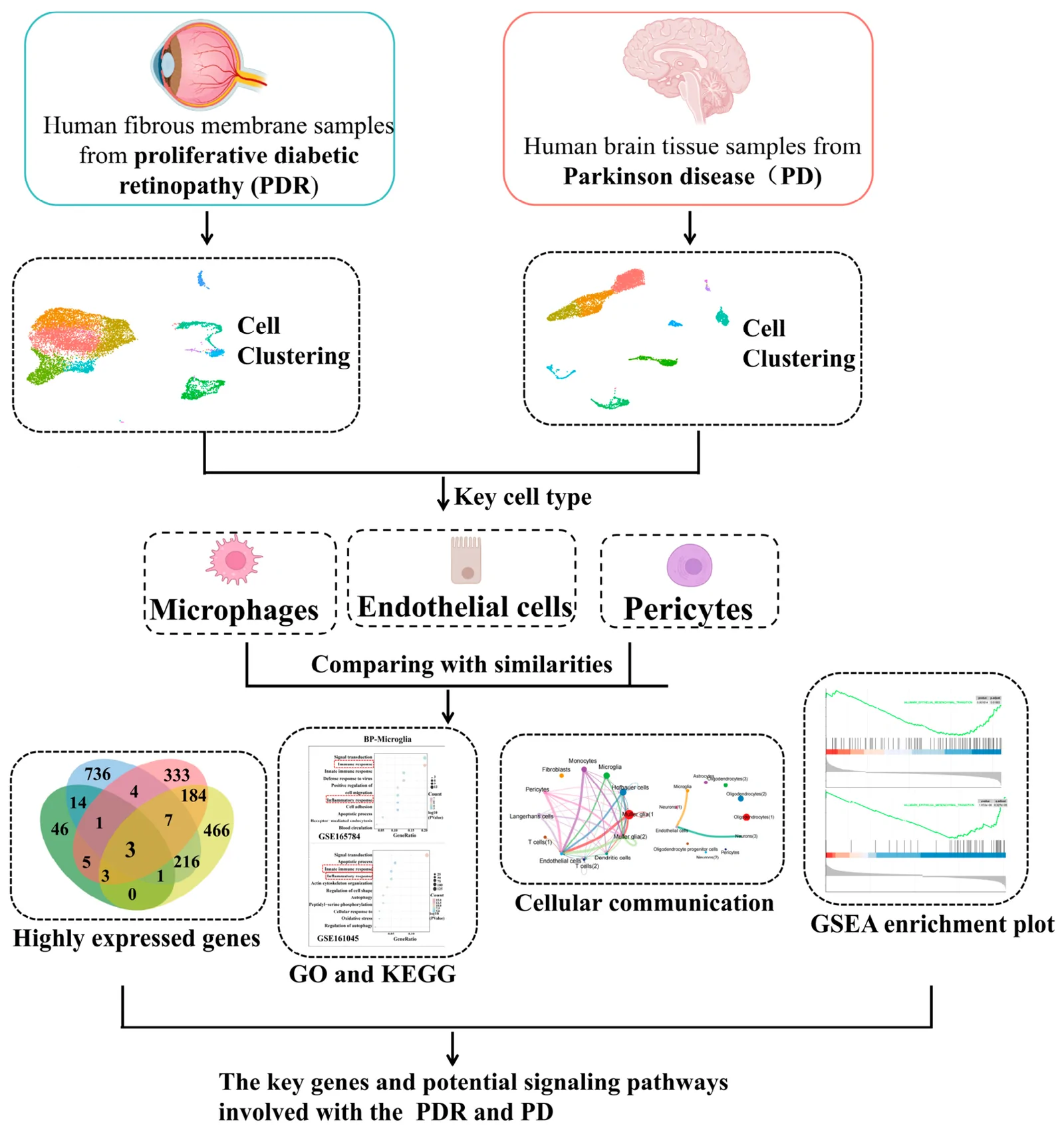
Overview of the study workflow. ScRNA-seq datasets from proliferative diabetic retinopathy (PDR) and Parkinson’s disease (PD) were obtained from the Gene Expression Omnibus (GEO) database. Key cell populations were identified through FindMarkers analysis, followed by GO and KEGG to characterize biological function in core cell types. Intercellular communication networks and major signaling pathways were inferred using CellChat. In addition, gene set enrichment analysis (GSEA) and gene set variation analysis (GSVA) were conducted to reveal critical functional pathways underlying shared pathogenic mechanisms.

Building upon these methodological advances, single-cell RNA sequencing (scRNA-seq) datasets obtained from PDR fibrotic membranes and PD brain tissues were employed in a comprehensive analysis (Figure 1). In the analyzed dataset, we identified three core cell types: microglia, ECs and pericytes. These cells served as pivotal units for immune surveillance and vascular regulation within the retina and central nervous system. Notably, A2M, NRP1, and ETS2, which are critical for understanding processes related to cell proliferation, neuroinflammation, and angiogenesis, were identified as key genes. Surprisingly, the ITGB2 signaling pathway was instrumental in facilitating cell-to-cell communication between microglia and ECs in both PDR and PD, known for its involvement in immune surveillance within neurovascular niches [19]. Overall, these insights underscored the importance of exploring the immune and vascular mechanisms underlying PDR and PD, as they may yield promising therapeutic targets for the effective management and treatment of these complex conditions.

## 2. Materials and Methods

### 2.1. Data Acquisition and Quality Control

Two scRNA-seq datasets (GSE165784 and GSE161045) were downloaded from the Gene Expression Omnibus (GEO) database. GSE165784 provided human fibrovascular membranes (FVMs) derived from five patients with PDR, who had not received preoperative anti-VEGF injections or retinal laser photocoagulation. Detailed sample information can be found in Table 1. Cells meeting the criteria of nCount_RNA > 500, nFeature_RNA < 5000, and mitochondrial gene content < 30% were filtered using the R software (version 4.3.2) integrated with the Seurat package (version 4.4.0) for subsequent analytical procedures. Meanwhile, GSE161045 provided human brain tissue samples isolated from four patients diagnosed with PD. For the PD-related datasets, quality control filtering was performed with parameters nCount_RNA < 15,000, 500 < nFeature_RNA < 2500, and mitochondrial gene content < 5%. The two datasets were analyzed separately and then compared at the level of matched cell populations. No matched healthy control samples were included in the source datasets used for the present analysis. Because the datasets were generated from different tissues and independent studies, we did not treat cross-dataset differences as disease-versus-control effects and did not perform biological batch correction that would artificially remove tissue-specific variation. Instead, dataset-specific normalization and clustering were performed separately, followed by comparison of shared cell-type transcriptional signatures.

### 2.2. Cell Clustering and Comparison

All samples were normalized using the SCTransform framework in Seurat (version 4.4.0), and the 2000 most variable genes were selected for principal component analysis (PCA). Clustering was performed separately for each dataset to avoid forcing cells from distinct tissues into a common expression space. Cell identities were assigned using the Cell Markers database and literature-reported markers [20,21]. Because GSE165784 and GSE161045 were generated independently and represent different tissues, no claim of complete technical batch removal or direct biological equivalence between the datasets was made.

### 2.3. Cell Composition and Cell-Type-Enriched Genes

The total number and proportion of cells were calculated in the entire dataset and within each specific group according to “cell. num”, “cell. freq” function. For cell-type characterization, the FindMarkers function (genes meeting an absolute fold-change threshold > 0.25 and adjusted *p*-value < 0.05 considered statistically significant) was used to identify genes enriched in each annotated cell population relative to the remaining cell populations within the same dataset. We further conducted a comparative analysis of cell-type-enriched genes within the same cell clusters across two datasets. Venn diagrams and dot plots were employed to visualize overlapping genes and different molecular signatures.

### 2.4. Functional Enrichment Analysis

To explore the biological functions of core cell populations in PDR and PD disease, we conducted Gene Ontology (GO) and Kyoto Encyclopedia of Genes and Genomes (KEGG) pathway enrichment analysis. The top 200 highly expressed genes were subsequently converted into their corresponding Entrez gene IDs for enrichment analysis via the R package “ClusterProfiler” (version 4.10.0).

### 2.5. Cell–Cell Communication Analysis

A comprehensive analysis of cellular communication was conducted on preprocessed scRNA-seq datasets via the “CellChat” function (version 1.6.1). Ligands or receptors with elevated expression levels were screened within distinct cell populations, after which gene expression profiles were mapped onto a protein–protein interaction (PPI) network. The intensity of intercellular communication was deduced by computing the communication probabilities of ligand–receptor pairs corresponding to each individual signaling pathway. The constructed intercellular communication network revealed prominent sender cells, receiver cells, mediator molecules, and influential factors, while the distribution of signaling gene expression was visualized using violin plots.

### 2.6. Gene Set Enrichment Analysis and Gene Set Variation Analysis

Gene set enrichment analysis (GSEA) is a widely applied tool in genomics research, facilitating the identification of biological pathways, functional annotations, and molecular signatures linked to distinct phenotypes or experimental settings. We retrieved the corresponding gene set (h.all.v7.4.symbols.gmt) and conducted GSEA analysis (version 1.64.0). Conversely, gene set variation analysis (GSVA) offers a more robust characterization of gene set variation across samples by converting gene expression profiles into gene set activity scores, which are independent of data ranking. For GSVA implementation (version 1.50.0), the mean expression levels of genes within each cell cluster were computed and matched with the relevant gene sets. Ultimately, the results of the GSVA were visualized through the construction of a heatmap.

## 3. Results

### 3.1. Identifying Cell Types in DR and PD

**Figure 2:**
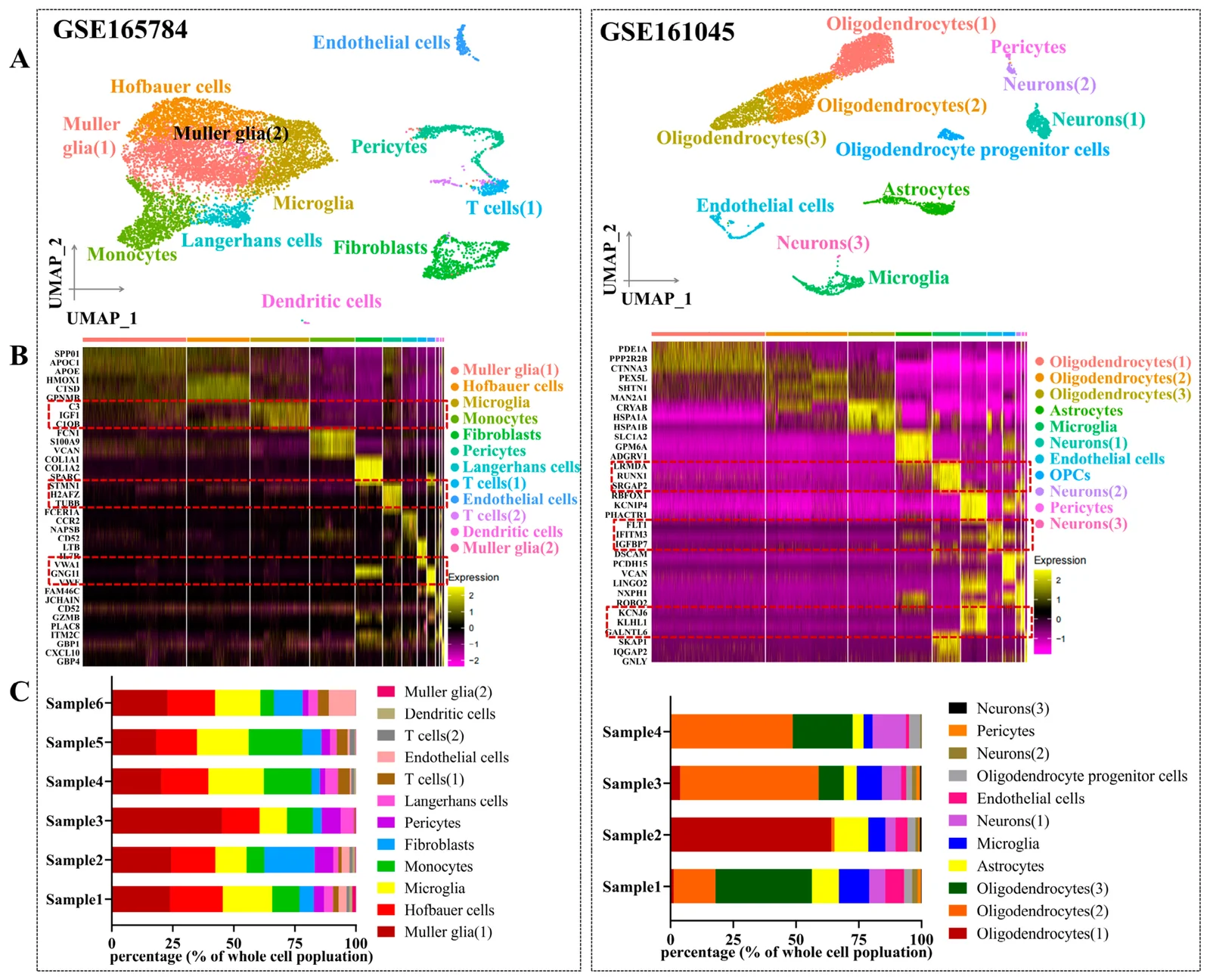
Single-cell atlas and cellular composition of PDR and PD brains. (**A**) UMAP visualization of major retinal and brain cell populations identified from the two scRNA-seq datasets. (**B**) Heatmaps showing representative marker gene expression across annotated clusters in each dataset. The marker genes in T cells, macrophages and endothelial cell are highlighted. (**C**) Bar plots presenting the proportional distribution of each cell type across individual samples, illustrating inter-sample variability and overall cellular composition in PDR (left) and PD (right).

After quality control and cell filtering, 23,437 genes from the PDR dataset, and 30,172 genes from the PD dataset were retained for subsequent in-depth analysis. Following normalization and dimensionality reduction, cells from the two datasets were classified into 12 and 11 distinct cell clusters, respectively. Uniform Manifold Approximation and Projection (UMAP) was employed to visualize the cellular clustering patterns (Figure 2A). Comparative assessment of both datasets highlighted microglia, ECs and pericytes as key populations of interest. A heatmap was then constructed to identify the marker genes for each cell cluster. As shown in Figure 2B, microglia were enriched in C3, IGF1, C1QB, LRMDA, RUNX1, and SRGAP2. ECs exhibited high expression levels in VWA1, GNG11, VWF, FLT1, IFITM3, and IGFBP7, and pericytes were characterized by STMN1, H2AFZ, TUBB, KCNJ6, KLHL1, and GALNTL6. Analysis of cell type proportion distribution across samples revealed that microglia, ECs, and pericytes accounted for approximately 11–30% of the total cell population in PDR patients, whereas their proportion ranged from 10% to 20% in PD brain tissue (Figure 2C). These findings indicate a substantial representation of these populations and suggest their potential involvement in vascular remodeling, neuroinflammation, and immune-regulatory pathways relevant to disease progression.

### 3.2. Comparing Cell-Type-Enriched Genes in the Core Cell Cluster Between PDR and PD

**Figure 3:**
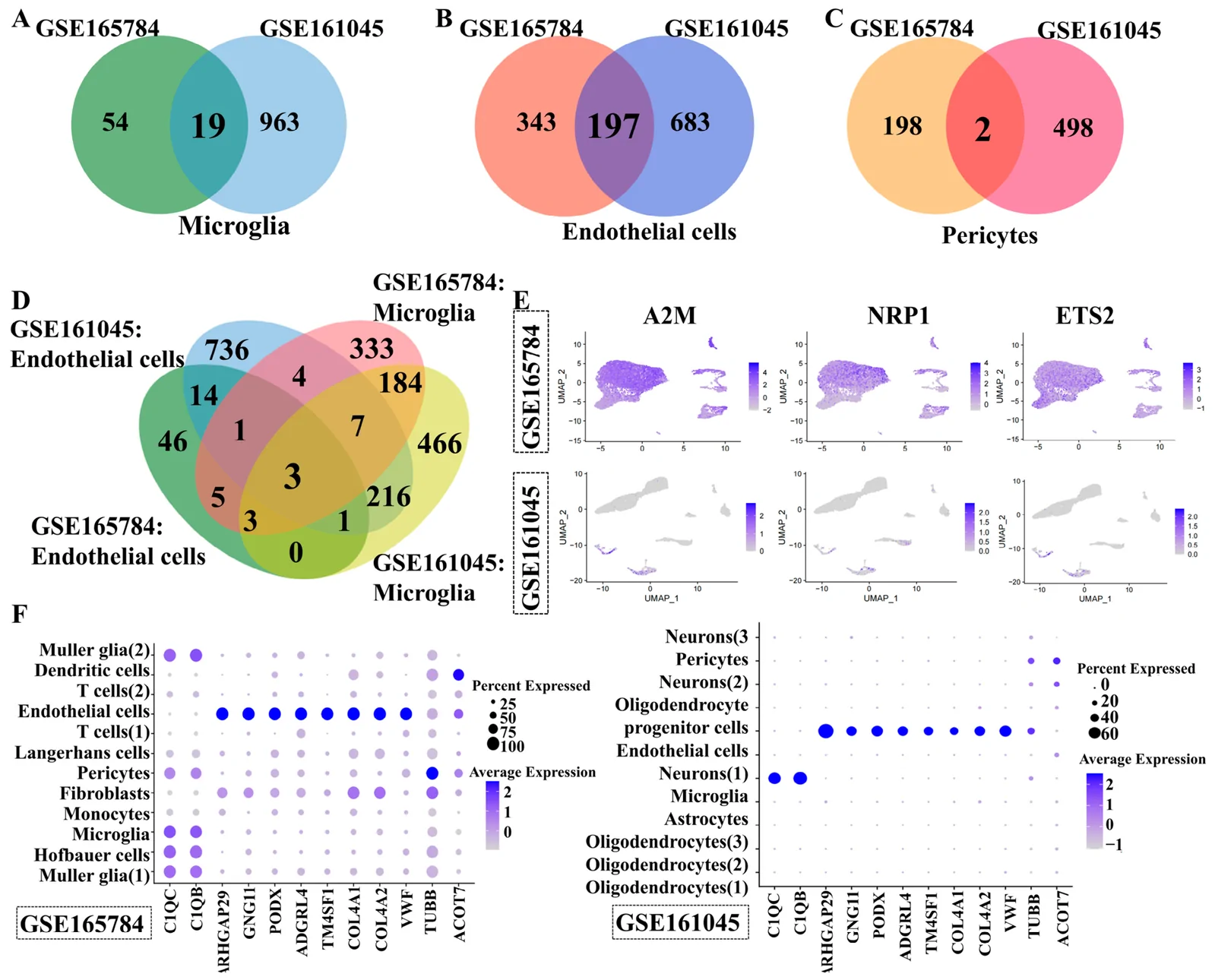
Identification of shared highly expressed genes between PDR and PD across key cell types. (**A**–**C**) Venn diagrams showing the overlap of genes in microglia (**A**), endothelial cells (**B**), and pericytes (**C**) between the GSE165784 and GSE161045 datasets. (**D**) Four-way Venn diagram illustrating the intersecting genes across microglia and endothelial cells from both datasets. (**E**) UMAP feature plots displaying the expression patterns of three shared core genes (A2M, NRP1, and ETS2) in microglia and endothelial cells across the two datasets. (**F**) Dot plots exhibiting the distribution and average expression levels of the selected genes across major cell types in GSE165784 (left) and GSE161045 (right).

To elucidate shared molecular features between PDR and PD, we conducted differential expression analyses of microglia, ECs and pericytes across both datasets. In dataset 1, 814 highly expressed genes were identified, including microglia (74), ECs (540) and pericytes (200). Meanwhile, dataset 2 exhibited a total of 2362 deregulated genes, including microglia (982), ECs (880) and pericytes (500). The Venn diagram demonstrated that 19 genes were common to both datasets in microglia. In addition, 197 and 2 genes were overlapped in ECs and pericytes, separately (Figure 3A). These findings provided an initial framework for identifying core pathogenic mediators common to PDR and PD. Notably, A2M, NRP1 and ETS2 were found in microglia and ECs within both datasets (Figure 3B). The UMAP showed that A2M, NRP1 and ETS2 were mainly expressed in microglia and ECs (Figure 3C). Dot plot analysis further highlighted cell-type-specific transcriptional signatures: C1QC and C1QB were highly enriched in microglia; ECs showed marked upregulation of ARHGAP29, GNG11, PODXL, ADGRL4, TM4SF1, COL4A1, VWF, and COL4A2; and pericytes exhibited prominent expression of TUBB and ACOT7 (Figure 3D). Focusing on these key genes may provide insight into their potential roles in the PDR and PD development within these core cell clusters.

### 3.3. GO and KEGG Analysis of Highly Expressed Genes in Microglia, Endothelial Cells and Pericytes

**Figure 4:**
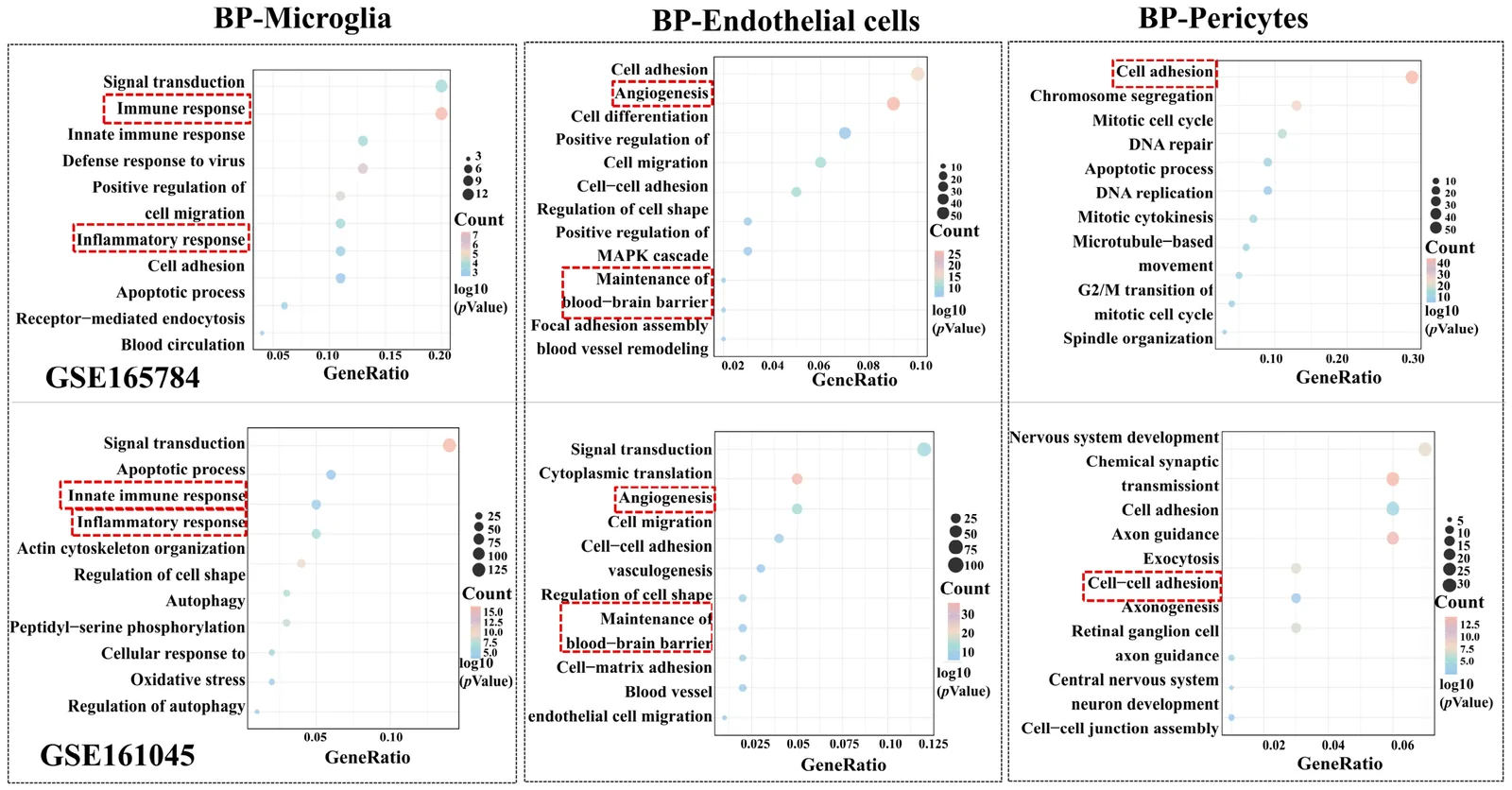
GO analysis of highly expressed genes in microglia, endothelial cells and pericytes. The (**upper**) panel is GSE165784 and the (**lower**) panel represents GSE161045.

To decipher the functional roles, regulatory mechanisms of highly expressed genes, and their potential interaction networks in PDR and PD, a comprehensive functional enrichment analysis was performed within microglia, ECs and pericytes across the two datasets. GO biological process (BP) analysis revealed that microglia were significantly enriched in “immune response” (*p* = 2.99 × 10^−8^, *p* = 8.17 × 10^−6^), “inflammatory response” (*p* = 6.95 × 10^−3^, *p* = 3.40 × 10^−8^), and “signal transduction” (*p* = 4.47 × 10^−3^, *p* = 3.61 × 10^−18^) (Figure 4: BP-Microglia). In Figure 4: BP-Endothelial cells, the enrichment of ECs was closely associated with “angiogenesis” (*p* = 2.16 × 10^−26^, *p* = 3.33 × 10^−13^), “cell–cell adhesion” (*p* = 1.52 × 10^−10^, *p* = 2.09 × 10^−4^), and “maintenance of blood–brain barrier” (*p* = 1.50 × 10^−6^, *p* = 5.35 × 10^−9^). Next, pericytes were observed with “cell adhesion” (*p* = 4.75 × 10^−48^, *p* = 3.88 × 10^−5^). Meanwhile, “nervous system development” (*p* = 6.64 × 10^−8^) and “central nervous system neuron development” (*p* = 9.79 × 10^−3^) were closely related to PD brain tissues (Figure 4: BP-Pericytes). Collectively, immune-inflammatory activation and vascular adhesion may provide critical functional evidence for the common and distinct pathogenic mechanisms.

**Figure 5:**
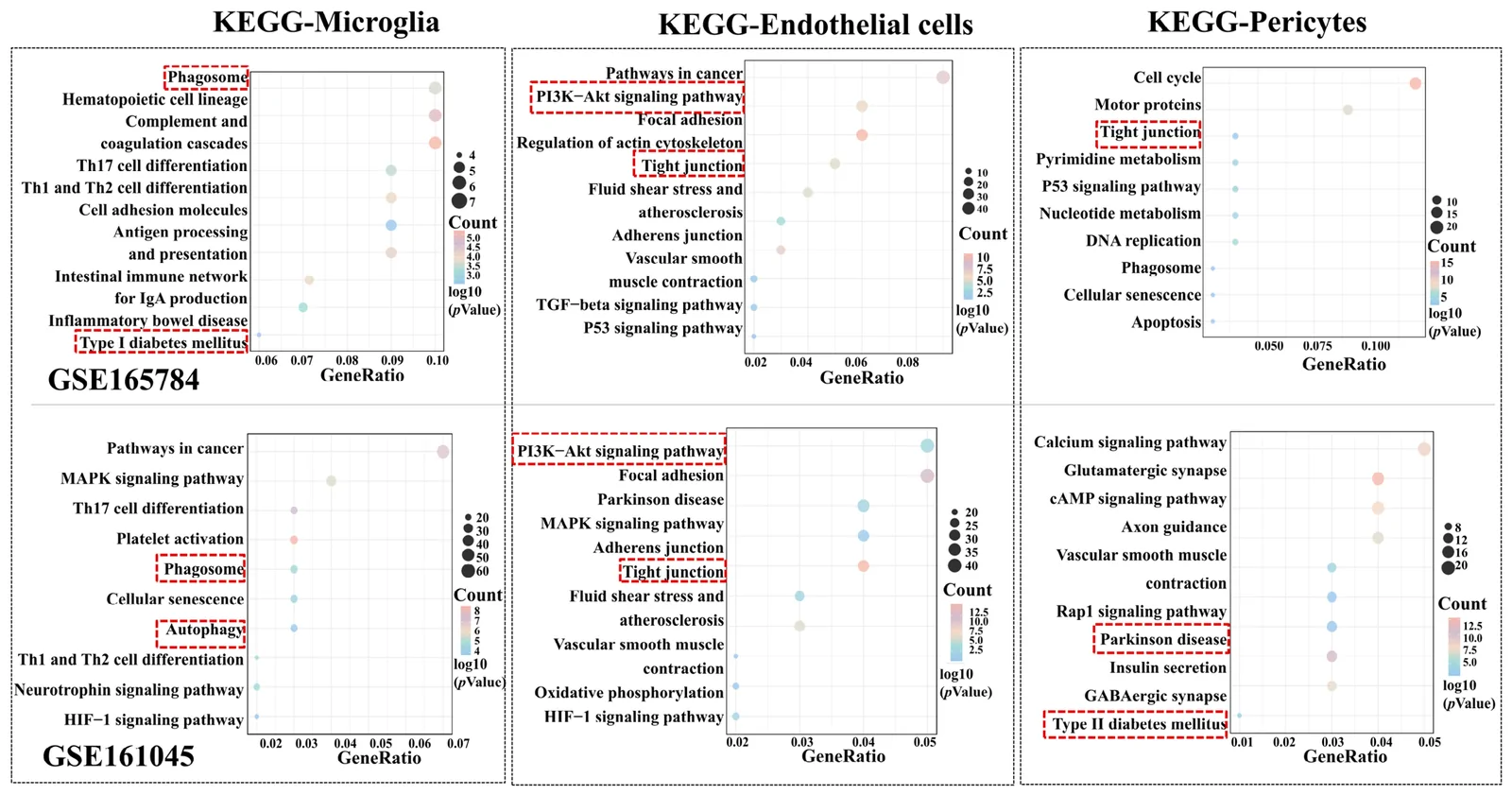
KEGG analysis of highly expressed genes in microglia, endothelial cells and pericytes. The (**upper**) panel is GSE165784 and the (**lower**) panel represents GSE161045.

KEGG pathway analysis provided additional insight into the molecular pathways underlying these shared mechanisms. Microglia were enriched in “Phagosome” (*p* = 1.92 × 10^−3^, *p* = 1.67 × 10^−5^); “Type I diabetes mellitus” (*p* = 1.66 × 10^−3^), and “Autophagy” (*p* = 1.31 × 10^−3^) (Figure 5: KEGG-Microglia), which may jointly contribute to retinal and neuronal degeneration. As shown in Figure 5: KEGG-Endothelial cells, ECs were mainly enriched in “PI3K-Akt signaling pathway” (*p* = 3.32 × 10^−7^, *p* = 4.03 × 10^−5^); “Tight junction” (*p* = 7.79 × 10^−7^, *p* = 5.84 × 10^−5^), and “Focal adhesion” (*p* = 6.30 × 10^−13^, *p* = 1.96 × 10^−12^). Then, pericytes were related to “Cell cycle” (*p* = 2.21 × 10^−18^) and “Tight junction” (*p* = 0.01) in dataset 1, while in dataset 2, it was found that pericytes were strongly relevant to “Insulin secretion” (*p* = 1.30 × 10^−9^) and “Type II diabetes mellitus” (*p* = 8.01 × 10^−4^) (Figure 5: KEGG-Pericytes). Taken together, these findings offered a deeper understanding of microglia’s, ECs’ and pericytes’ functional implications in the analyzed datasets and delineate a coherent functional landscape in the PDR and PD development.

### 3.4. Elucidating ITGB2-Mediated Crosstalk Between Microglia and Endothelial Cells in PDR Fibrovascular Membranes

**Figure 6:**
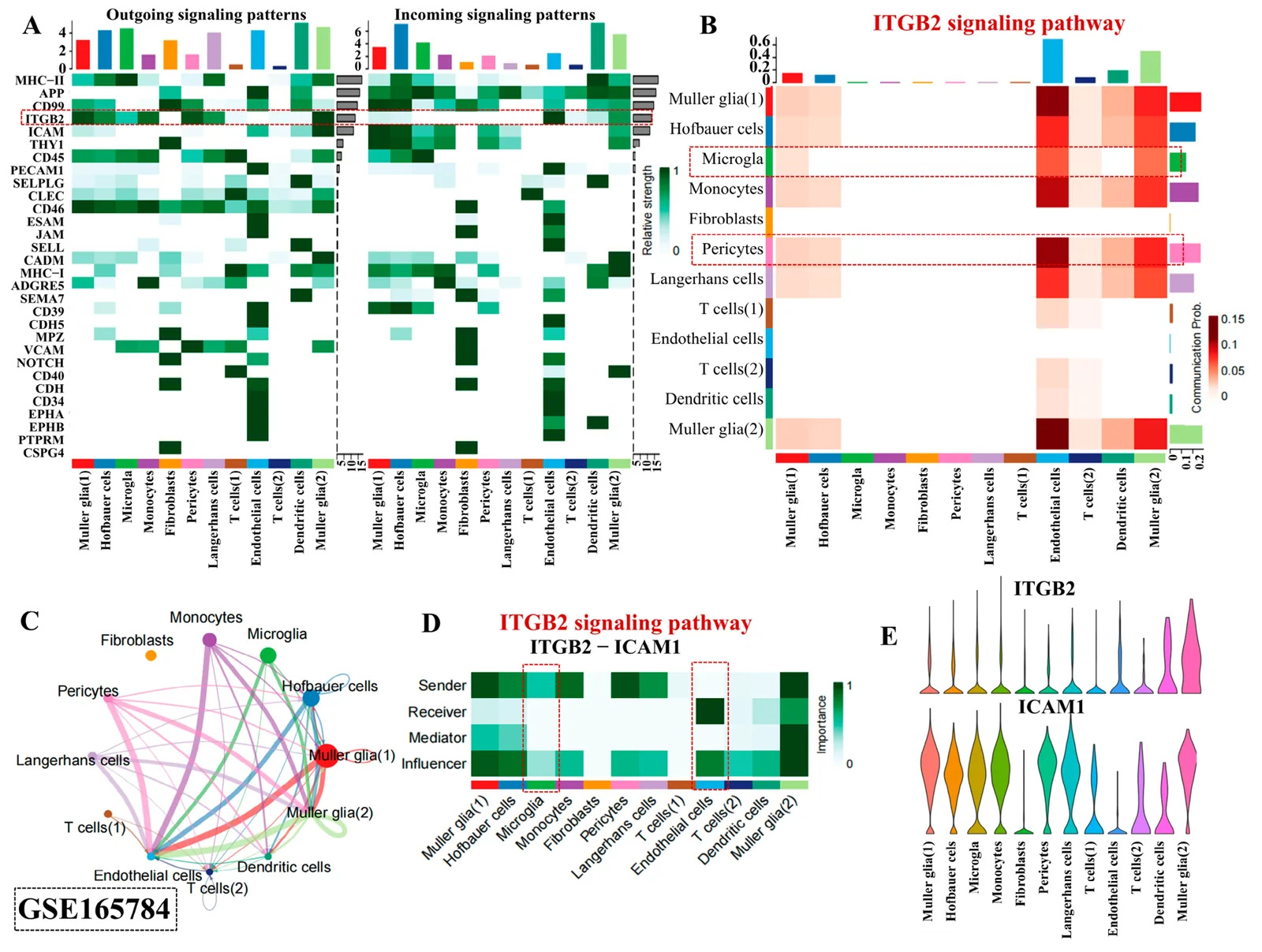
Cell–cell communication analysis highlighting the ITGB2 signaling pathway in PDR datasets. (**A**) Heatmaps showing outgoing (left) and incoming (right) signaling patterns across major cell types, with ITGB2-related interactions highlighted. (**B**) Heatmap of the ITGB2 signaling pathway, illustrating communication probabilities between sender and receiver cell populations. (**C**) Circle plot depicting the overall ITGB2-mediated communication network among key cell types in the GSE165784 dataset. (**D**) Heatmap showing sender, receiver, mediator, and influencer roles of major cell types within the ITGB2–ICAM1 signaling axis. (**E**) Violin plots displaying ITGB2 and ICAM1 expression levels across individual cell clusters.

Notably, ITGB2 signaling had emerged as a key player in the signaling dynamics within PDR (Figure 6A). Furthermore, this signaling cascade, originating from ECs, was predominantly received by various cellular populations in the FVM of PDR (Figure 6B). An intriguing aspect of the ITGB2 signaling pathway was its reliance on paracrine signaling mechanisms, which facilitated intercellular interactions and enhanced communication between cells (Figure 6C). Further analysis of the network centrality within the inferred ITGB2 signaling interactions (ITGB2–ICAM1), revealed that pericytes predominantly serve as signaling senders, exerting significant influence over both microglia and ECs (Figure 6D). Additionally, as illustrated in Figure 6E, ITGB2 was notably expressed in ECs and played an essential role in Müller glial function and signaling, while ICAM1 was involved in processes related to microglia and Langerhans cells. Collectively, these comprehensive findings strongly suggested that the ITGB2 signaling network enhanced intricate cellular communication between microglia and ECs, contributing significantly to the pathogenesis and progression of PDR.

### 3.5. Regulation of ITGB2 Signaling Pathway Complexity Involving Microglia and Endothelial Cells in PD

**Figure 7:**
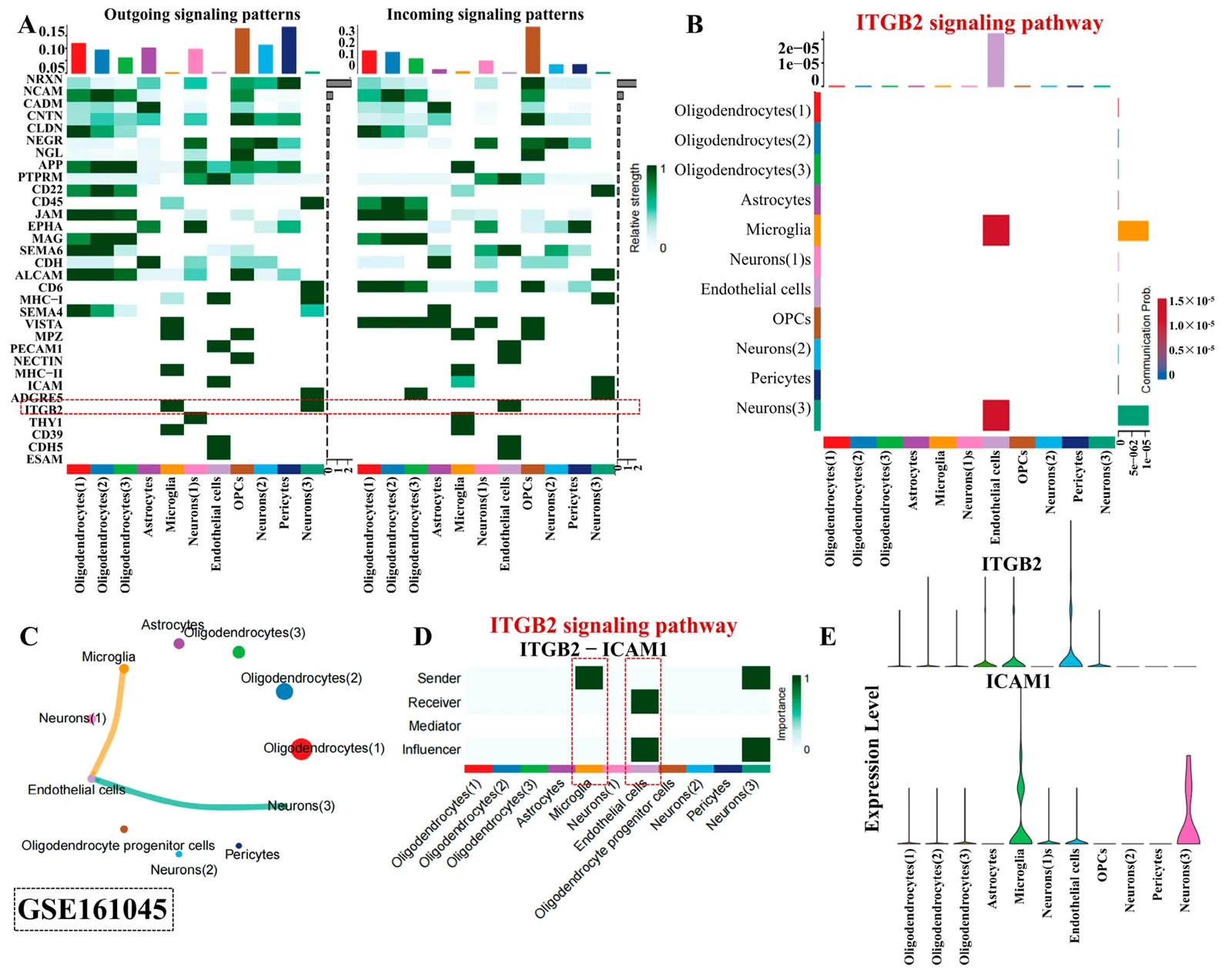
Cell–cell communication analysis highlighting the ITGB2 signaling pathway in PD datasets. (**A**) Heatmaps showing outgoing (left) and incoming (right) signaling patterns across major cell types, with ITGB2-related interactions highlighted. (**B**) Heatmap of the ITGB2 signaling pathway, illustrating communication probabilities between sender and receiver cell populations. (**C**) Circle plot depicting the overall ITGB2-mediated communication network among key cell types in the GSE161045 dataset. (**D**) Heatmap showing sender, receiver, mediator, and influencer roles of major cell types within the ITGB2–ICAM1 signaling axis. (**E**) Violin plots displaying ITGB2 and ICAM1 expression levels across individual cell clusters.

To gain a deeper understanding of the communication network between cells in the brain tissue affected by PD and to compare it with the FVM in PDR, we investigated the intercellular interactions and associated signaling pathways. Figure 7A underscored the crucial role of the integrin beta-2 (ITGB2) pathway in PD pathophysiology. Meanwhile, microglia emerged as the predominant source of the ITGB2 signaling pathway in ECs within PD brain tissue (Figure 7B). As shown in Figure 7C, microglia transmitted signals to ECs by paracrine means. Importantly, microglia acted as the principal senders, whereas ECs played predominant roles as the primary receivers and influencers, collectively governing the intricate communication dynamics within the ITGB2 signaling pathway (Figure 7D). Notably, ITGB2 demonstrated exclusive expression in ECs, while Intercellular Adhesion Molecule 1 (ICAM1) exhibited high expression levels in microglia and neurons (Figure 7E). Because ligand–receptor inference depends on transcript abundance and the curated interaction database, these results should be interpreted as a predicted communication relationship rather than direct evidence of functional ITGB2–ICAM1 signaling. Importantly, the directionality differed from that inferred in the PDR dataset, supporting a context-dependent rather than universally conserved sender–receiver configuration.

### 3.6. Distinct Functional Roles and Cross-Talk Between Microglia and Endothelial Cells in PDR and PD

**Figure 8:**
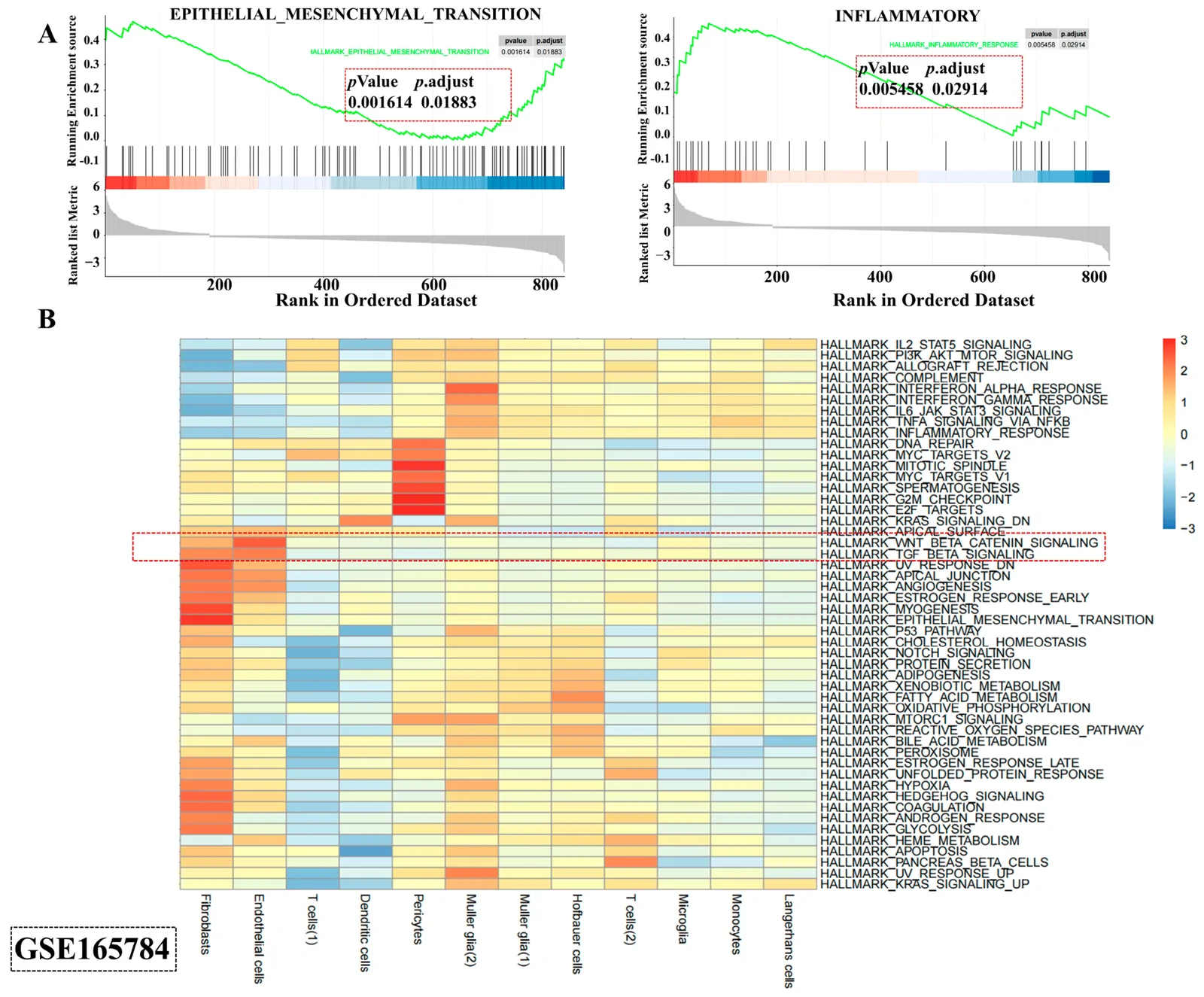
Unique biological functions and interactions of microglia and ECs in PDR. (**A**) GSEA plots for the “Epithelial_Mesenchymal_Transition” (left) and “Inflammatory Response” (right) pathways in PDR. (**B**) Heatmap of hallmark pathway activity scores across cell types. Different shades of color represent different intensities of expression.

**Figure 9:**
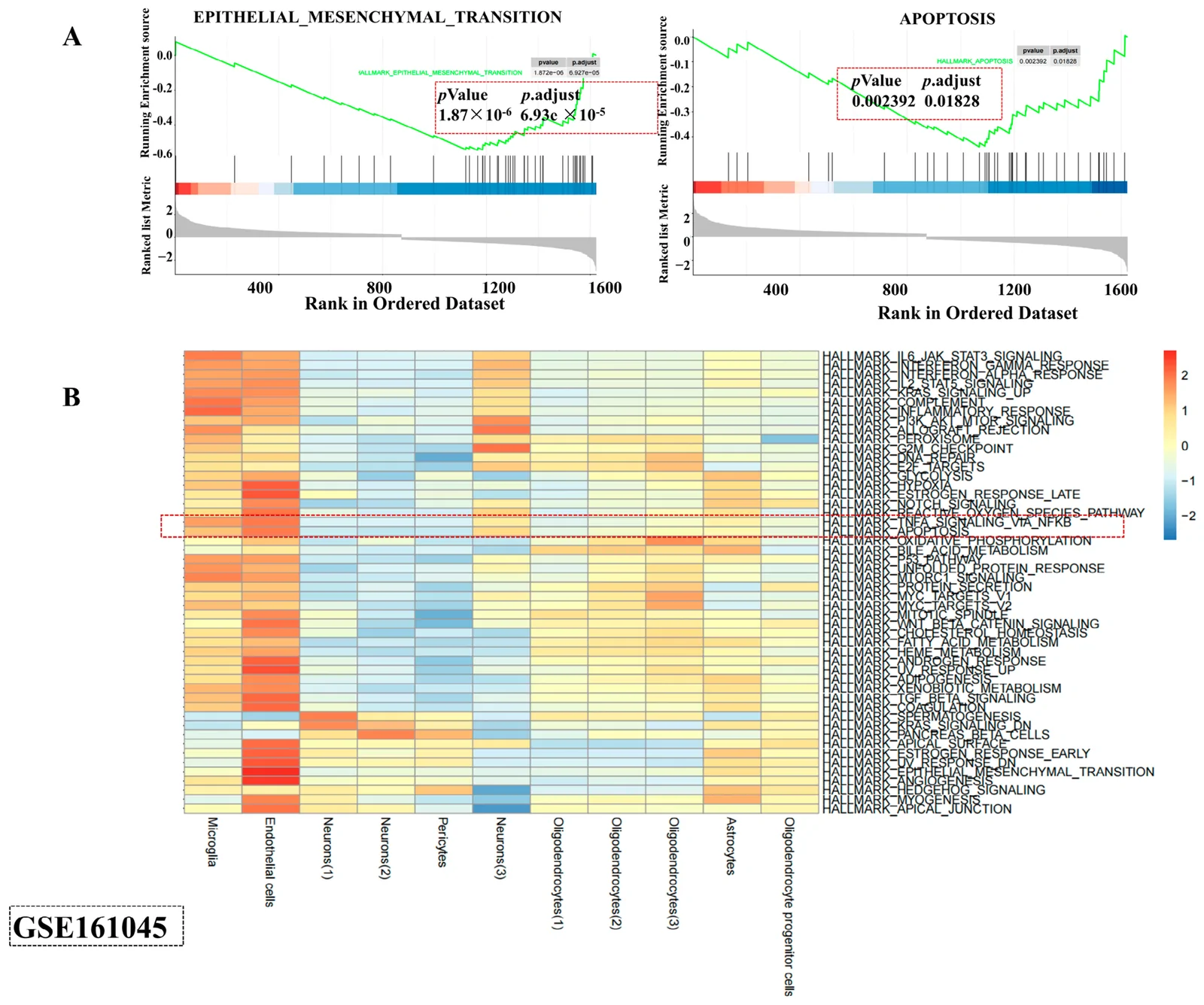
Unique biological functions and interactions of microglia and ECs in PD. (**A**) GSEA plots for the “Epithelial_Mesenchymal_Transition” (left) and “Apoptosis” (right) pathways in PD. (**B**) Heatmap of hallmark pathway activity scores across cell types. Different shades of color represent different intensities of expression.

GSEA and GSVA were employed to uncover the functional correlation between microglia and ECs in PDR and PD. Our findings revealed a compelling correlation between gene sets enriched in both datasets. As shown in Figure 8A and Figure 9A, we identified significant enrichments for several key pathways, including “Hallmark_Epithelial_Mesenchymal_Transition” (*p*_adj = 0.018, 6.927 × 10^−5^), “Inflammatory” (*p*_adj = 0.029), and “Apoptosis” (*p*_adj = 0.018). This shared enrichment highlighted potential common molecular pathways and biological functions that could be critical in both PDR and PD. Moreover, both microglia and ECs exhibited significant enrichment in pathways related to cell proliferation and inflammation response, particularly those mediated by WNT-beta and TGF-beta signaling (Figure 8B). Additionally, both cell types displayed enrichments in apoptosis, hypoxia, and the TNFA/NFκB signaling pathway (Figure 9B). Together, these analyses provided valuable insights into the roles of these cell populations in the pathogenesis of their respective diseases, enhancing our understanding of shared mechanisms and facilitating meaningful comparisons between PDR and PD. This knowledge could inform future therapeutic strategies aimed at mitigating disease progression through targeted modulation of these immune pathways.

**Table 1:** Sample information.

Dataset	Sample	Group	Tissue	Platform	Included	Reason
GSE165784	GSM5049904	PVR	Fibrous membrane	HiSeq X Ten ^1^	No	Different retinal disease
GSE165784	GSM5049905	PDR	Fibrous membrane	HiSeq X Ten	Yes	Principal PDR analysis
GSE165784	GSM5049906	PDR	Fibrous membrane	HiSeq X Ten	Yes	Principal PDR analysis
GSE165784	GSM5277737	PDR	Fibrous membrane	HiSeq X Ten	Yes	Principal PDR analysis
GSE165784	GSM5690478	PDR	Fibrous membrane	HiSeq X Ten	Yes	Principal PDR analysis
GSE165784	GSM5690479	PDR	Fibrous membrane	HiSeq X Ten	Yes	Principal PDR analysis
GSE161045	GSM4888887-890	Control	Striatum	NovaSeq 6000 ^2^	No	Not in principal PDR–PD comparison
GSE161045	GSM4888891-894	AD	Striatum	NovaSeq 6000	No	Different neurodegenerative disease
GSE161045	GSM4888895-898	PD	Striatum	NovaSeq 6000	Yes	Principal PD analysis

1: GPL20795 (USA); 2: GPL24676(USA).

## 4. Discussion

DR and PD are two widespread conditions that profoundly impact the quality of life worldwide. DR, a microvascular complication associated with diabetes, is characterized by progressive damage to the retinal vasculature, leading to potential blindness [22]. Meanwhile, PD is a neurodegenerative disorder primarily affecting motor functions, but it also manifests with non-motor symptoms [23]. Recent research highlights a complex interplay between DR and PD, highlighting shared pathophysiological mechanisms, particularly in neuroinflammation and vascular dysregulation [24,25]. Optical coherence tomography (OCT) has emerged as an invaluable tool for visualizing and monitoring retinal alterations associated with both DR and PD [26,27], providing a non-invasive and early method for assessing disease progression. The parallels in the pathophysiology of DR and PD have sparked interest in further exploring the underlying pathways of disease progression. Gaining a deeper understanding of these relationships is crucial for developing therapeutic strategies that can effectively address both conditions. In our study, we identified key cellular populations and their dynamic communication patterns, shedding light on the overlapping biological pathways that contribute to the progression of these diseases. These findings are essential for advancing interventions that may enhance patient outcomes in both DR and PD.

The interactions among microglia, ECs and pericytes in both DR and PD highlighted the critical role of immune–vascular communication in driving dysfunction within the neurovascular unit. Previous studies have emphasized the role of microglia in mediating local inflammatory responses and contributing to vascular permeability changes in both retinal and cerebral contexts [28,29]. ECs played a crucial role in maintaining barrier integrity, regulating angiogenesis, and facilitating leukocyte transmigration [30]. Meanwhile, pericytes were essential for stabilizing capillaries and modulating basement membrane composition. While the literature has documented the individual contributions of these cell types to disease pathogenesis, recent single-cell analyses suggested that their concerted interactions were necessary for the propagation of chronic inflammation and vascular compromise [31]. The present findings reveal that microglial-derived cytokines and chemokines stimulate endothelial and pericyte dysfunction, initiating a feedforward loop that perpetuates neurodegeneration and microvascular pathology. This contrasts with earlier models that prioritized neuronal or endothelial dysfunction as primary events. In addition, the emergent properties of immune–vascular cellular networks may offer novel interventional targets.

Through the comparison and analysis of highly expressed genes across two datasets, we identified overlapping transcriptional features between PDR FVM and PD brain tissue. Notably, A2M, NRP1, and ETS2 were consistently expressed in both microglia and ECs, indicating their potential involvement in the pathogenesis of these two conditions. A2M, a well-known broad-spectrum protease inhibitor, played a critical role in modulating neuroinflammation and facilitating extracellular matrix remodeling [32]. A previous study demonstrated that inhibiting SOCS3–STAT3 and TIMP1–A2M pathways could reduce DR-related pathological damage [33]. Moreover, a clinical study firstly revealed the increased level of A2M in the tears of patients with PDR [34], further underscoring its relevance in ocular pathology. Additionally, a meta-analysis indicated that the rs669 (A/G) polymorphisms in the A2M gene were associated with increased risk in PD [35]. NRP1, a multifunctional co-receptor, was shown to modulate VEGF-A-mediated signaling to migration, survival and three-dimensional sprouting of ECs [36]. Recent studies found that targeting the Sema3A/NRP1 pathway could address treatment limitations in DR [37]. Furthermore, lncRNA DLX6-AS1 has been implicated in enhancing microglial inflammatory responses in PD by regulating the miR-223–3p/NRP1 axis [38]. These findings signified a mechanistic intersection between ocular and cerebral microvasculature dysfunction. ETS2 was a transcription factor and governed cellular stress responses and apoptosis. Its activation has been linked to exacerbating DR by promoting EC proliferation and inflammatory responses [39]. Although research on the correlation between ETS2 and PD is still limited, the activation of ETS2 by oxidative stress has been shown to induce Bcl-xL expression, contributing to glial cell survival in amyotrophic lateral sclerosis (neurodegenerative disorder) [40]. Together, these findings illustrate a mechanistic intersection between ocular and cerebral microvasculature dysfunction, suggesting that shared molecular pathways may underlie the pathogenesis of both DR and PD, warranting further exploration for therapeutic interventions.

The predicted importance of the ITGB2 (ITGB2–ICAM1) signaling pathway in mediating communication between microglia and ECs presented a compelling mechanism for cross-talk in DR and PD. ITGB2, encoding the β2 integrin subunit, was central to leukocyte adhesion, migration, and the orchestration of immune surveillance within neurovascular niches. Evidence suggested that the FAK/PI3K/AKT signaling pathway was suppressed upon ITGB1 silencing to alleviate the progression of DR [41]. ICAM1 was recognized as a key adhesion molecule during the recruitment of leukocytes and participated in a variety of physiological processes, including T cell regulation, macrophage polarization, cellular migration, metastasis and cancer development [42]. A meta-analysis showed elevated ICAM1 levels in patients with DR, correlating with disease severity [43]. The increased presence of ICAM1 was strongly associated with compromised microvascular function and heightened oxidative stress in diabetes pathology [44]. Moreover, research indicated that ICAM1 may contribute to the degeneration of dopaminergic neurons by regulating inflammatory responses, as observed in MPTP-induced PD mouse models [45]. The current findings expand upon these paradigms by demonstrating that ITGB2-driven microglial–EC communications are not merely secondary consequences but may constitute primary pathogenic drivers in both DR and PD.

In this study, the GSVA and GSEA analyses offered robust evidence supporting the biological correlation between microglia and ECs in both DR and PD. These findings elucidated the interconnectedness of these cell types by identifying shared enriched gene sets and emphasizing their roles in apoptosis, immune modulation, inflammation and epithelial–mesenchymal transition (EMT). Furthermore, the significant involvement of WNT-beta and TGF-beta signaling in regulating cell proliferation and inflammatory responses underscored the intricate dynamics between microglia and ECs. By revealing these shared biological functions, our analyses enhance the understanding of how immune and vascular alterations contribute to the pathogenesis of DR and PD, paving the way for future therapeutic strategies that target these pathways to mitigate disease progression. Overall, this study provides an exploratory framework for identifying potentially shared molecular features between PDR and PD. Rather than establishing a common pathogenic mechanism, our findings generate testable hypotheses regarding immune–vascular interactions. Additionally, our research has some limitations, including the small number of biological donors (six PDR and four PD patients), different tissue origins, independent experimental platforms, potential technical batch effects, lack of matched healthy controls, and the need for independent dataset and experimental validation. We propose that future studies should validate our findings through experimental validation in animal models of DR and PD, clinical cohort studies with longitudinal sampling, and functional studies (e.g., knockout or overexpression of candidate genes in relevant cell types).

## 5. Conclusions

In conclusion, this study provides an exploratory characterization of cellular and molecular features potentially shared between DR and PD. We focused particularly on the contributions of microglia, ECs and pericytes across retinal and brain tissues. The predicted ITGB2–ICAM1 interaction represents a candidate molecular connection identified through computational analysis and provides a hypothesis for further investigation. By delineating cellular heterogeneity and transcriptional alterations common to both disorders, this work lays a foundation for more targeted and mechanism-based therapeutic strategies. Continued investigation in this field will further refine disease management and support the development of precision interventions for DR and PD.

## Data Availability

The single-cell RNA-seq datasets analyzed in this study are publicly available in the Gene Expression Omnibus under accession numbers GSE165784 (PDR fibrovascular membrane dataset) and GSE161045 (PD brain tissue dataset). The analysis code and processed objects are available from the corresponding author upon reasonable request.

## Abbreviations

DR	Diabetic retinopathy
PD	Parkinson’s disease
scRNA-seq	Single-cell RNA sequencing
GSEA	Gene set enrichment analysis
GSVA	Gene set variation analysis
ECs	Endothelial cells
VEGF	Vascular endothelial growth factor
GEO	Gene Expression Omnibus
GO	Gene Ontology
KEGG	Kyoto Encyclopedia of Genes and Genomes
PPI	Protein–protein interaction
